# What makes general practice work: the role of continuity in efficient and sustainable primary care

**DOI:** 10.3399/BJGP.2025.0038

**Published:** 2025-07-22

**Authors:** Tom Marshall, Stefan Scholtes, Steven Wyatt, Patrick Burch, Sharon Spooner, Panagiotis Kasteridis, Lyvia de Dumast, Rosa Parisi, Harshita Kajaria-Montag, Geraldine Clarke, Laura Jefferson, Brian Willis

**Affiliations:** 1 Institute of Applied Health Research, University of Birmingham, Birmingham, UK; 2 Professor of Health Management, Judge School of Business, University of Cambridge, Cambridge, UK; 3 Senior Analyst, Strategy Unit, NHS Midlands and Lancashire Commissioning Support Unit, Stoke-on-Trent, West Bromwich, UK; 4 NIHR Clinical Lecturer, School of Health Sciences, University of Manchester, Manchester, UK; 5 Honorary Clinical Lecturer, School of Health Sciences, University of Manchester, Manchester, UK; 6 Senior Research Fellow, Centre for Health Economics, University of York, York, UK; 7 PhD student, Institute of Applied Health Research, University of Birmingham, Birmingham, UK; 8 Research Fellow, School of Health Sciences, University of Manchester, Manchester, UK; 9 Assistant Professor of Operations, Kelley School of Business, Indiana University, Bloomington, USA; 10 Senior Analytical Manager, The Health Foundation, London, UK; 11 Associate Professor, Department of Health Sciences, University of York, York, UK; 12 Clinical Senior Lecturer, Institute of Applied Health Research, University of Birmingham, Birmingham, UK

## Introduction

Relational continuity of care in general practice has been declining for many years, largely because of increases in general practice size and in part-time working by GPs. Data show continuity is lower in larger practices, which tend to have more GPs, in those with a higher proportion of part-time GPs, and in those that are more reliant on temporary staff.^1–3^ Arguments in favour of relational continuity of care in primary care often emphasise its importance as a core value, that it is preferred by patients or for its effects on quality of care. There are also important pragmatic reasons for general practices to prefer higher continuity of care: efficiency and sustainability.

In economics, efficiency is the ratio of outputs produced per unit of input. General practices combine inputs (for example, staff, facilities, technology) to produce outputs in two key domains: clinical care and patient experience. They deliver these outputs through multiple processes (patient contacts, appointments, prescriptions issued, diagnostic tests requested, and so on). The quality and quantity of these processes determine general practices’ effectiveness in delivering high-quality clinical care and a positive patient experience.

Sustainability is the ability to meet the needs of the present without compromising the ability to meet future needs. In general practice, this means the ability to deliver patient experience and clinical care, without compromising the ability to deliver these in the future. Two key domains affect sustainability: staffing and financial balance. These are closely related as a general practice’s financial sustainability is affected by its ability to recruit and retain staff.

In the UK, around 7000 independent general practices, mainly owned by GP partners, deliver the majority of first-contact medical services. They provide initial and ongoing health care for their registered patients, including referrals to specialists. GP partners invest in practice premises and share profits or losses, while also being responsible for staffing and expenses. The NHS contracts these practices, reimbursing them per registered patient and through various allowances and incentive schemes such as the Quality and Outcomes Framework (QOF). Although health policymakers influence general practices through regulations and financial incentives, practices have a degree of independence and practices differ significantly in ethos, organisation, staffing, appointment systems, and operational methods. Practices therefore vary in their efficiency — the extent to which they deliver clinical care and patient experience within their resources — and vary in their sustainability, both in relation to staffing and finance.

We describe how relational continuity of care, the ongoing relationship between patients and clinicians, is a critical factor in supporting efficiency and sustainability.

## Clinical quality of care

Continuity of care is consistently linked to clinical quality, with better prescribing and markedly reduced antibiotic prescribing.^4,5^ It is associated with better health outcomes for many long-term conditions and with reduced unplanned admissions to hospital.^6,7^ Disruption of relational continuity leads to increased use of specialty, urgent, and emergency care.^8,9^ Increases in continuity of care lead to reduced unplanned admissions to hospital.^10,11^ In this domain, the role of continuity is unequivocal. Continuity improves clinical quality of care.

## Patient experience

Patient experience reflects how well practices meet patients’ subjective needs in relation to their expectations. Annual surveys assess patient experience and show it to be closely correlated with reported access to general practice. The strength of the correlation has increased over time (Pearson *R* = 0.75, *R* ^2^ = 0.57 in 2012; *R* = 0.91, *R* ^2^ = 0.81 in 2024). Access and patient experience are essentially the same characteristic. Both reported access and patient experience have consistently declined since 2011.^12^ What determines access and hence patient experience?

**Figure 1.**
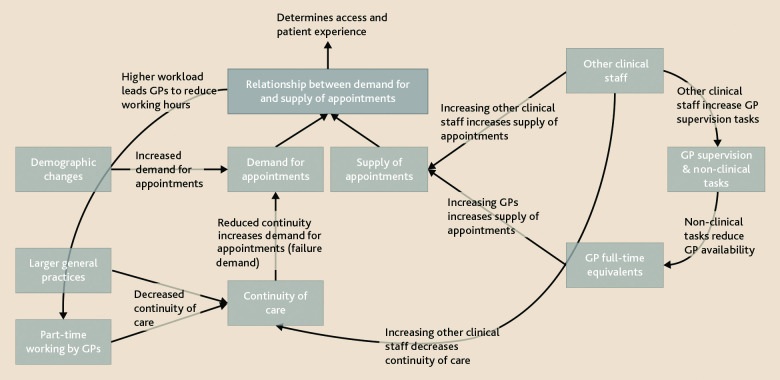
Factors influencing patient demand and supply of appointments in primary care.

Access is in large part determined by the balance between demand for and supply of appointments. Demand for appointments is driven by both external factors, such as population characteristics, morbidity prevalence, and access to secondary care, and internal factors, including continuity of care. Higher relational continuity is associated with lower demand for future appointments: patients who see their usual (most frequently consulted) GP require 18% fewer visits.^5,^ ^13^ Much of this advantage is maintained by seeing the second most frequently consulted GP.^14^ Influences on demand are shown in the left side of Figure 1. We have already noted that practices with higher continuity of care deliver higher clinical quality of care. We can now see that they do so through fewer appointments. In effect, by delaying the need for future consultations, continuity reduces failure demand, the need for repeat contact.^15^ If the supply of appointments is fixed, reduced demand for appointments is also likely to improve access and hence patient experience (top of Figure 1). This seems paradoxical. At the time when an individual requests an appointment, there is a trade-off between an immediate appointment with any GP and continuity with a specific GP. But because practices with high continuity have lower demand for appointments, we observe, counterintuitively, that they also tend to have better access.

Supply of appointments to meet demand is primarily dependent on the numbers of qualified clinical staff. However, staffing has changed in ways that have decreased continuity of care. From September 2015 to December 2024, numbers of full-time equivalent GPs declined 2%, while nurses increased 17% and other clinical staff increased by 428%, mainly since 2020.^16^ The decline in full-time equivalent GPs is partly driven, not only by younger GPs choosing to work part time but also by increasing GP turnover.^17,18^ The changing workforce reduces continuity in two ways: it reduces the proportion of appointments with GPs, fragmenting appointments between different clinical staff; it increases GPs’ supervisory responsibilities therefore reducing time for GP appointments. This is shown in the right side of Figure 1. But reduced continuity increases demand for appointments, therefore eroding potential gains in access through increasing supply of appointments. It is noticeable that there is a positive correlation between the number of GPs and patient experience, (that is, access), but a negative correlation with numbers of non-medical clinical staff.^19^ This may be because practices facing workload pressures and poor access respond by using more non-medical clinical staff (reverse causation) or because the additional supply of non-medical appointments is not improving access as it is offset by increased demand due to lower continuity. This merits further investigation.

*“Continuity of care enhances the efficiency with which general practices deliver patient experience and clinical quality of care.”*

## Staffing

GP satisfaction has gradually declined, with more GPs working part time and many expressing intentions to leave direct patient care. Factors contributing to GPs leaving include increased workload, emotional strain, and negative public sentiment.^17^ Intensity of workload is frequently cited as a reason for reducing working hours.^20^ The mismatch between demand for and supply of appointments contributes to GPs reducing their hours, reducing continuity of care and therefore contributing to increasing demand (Figure 1). Yet continuity of care is also important for GP satisfaction, by supporting relationships with patients and colleagues, giving meaning and joy to doctors’ work, and may play a key role in workforce sustainability.^21,22^ This is consistent with the observation that GP turnover is higher in larger general practices, which tend to have lower continuity.^23^ Further research would help quantify the contribution of continuity of care to staff recruitment and retention.

## Financial sustainability

Financial sustainability is a concern for general practices, with staff salaries being a significant cost. Practice closures are increasing, particularly for smaller practices in more deprived areas. Failure to recruit or retain GPs is cited as the main reason for practice closure, with funding a contributing factor.^24^ In a system where funding is not linked to clinical activity levels, continuity potentially contributes to financial sustainability in two ways. First, it reduces demand for appointments, therefore reducing staff workload without loss of income. Second, it potentially improves staff retention. However, there are few data and little research on the financial implications of continuity of care for general practices and this warrants further investigation.

## Conclusion

General practices deliver care in multiple domains. Continuity of care enhances the efficiency with which general practices deliver patient experience and clinical quality of care. Continuity of care may also contribute to general practice sustainability through its effects on staffing, although this is less explored.

Continuity is declining. We would anticipate this to result in higher general practice workload, declining access, and therefore poorer patient experience. We would expect it to result in more unplanned admissions to hospital. We would expect it may contribute to problems in staffing general practices and general practices’ sustainability. All of these anticipated effects are visible.

Why has continuity declined? Some of the downward pressure on continuity of care is the result of increasing demand for appointments, itself due to demographic change, an ageing population, and increasing multimorbidity. Some is due to societal changes, including a growing preference for part-time working among GPs. Some is due to growth in practice size. Some is due to specific policies: growing use of non-GPs in primary care, fragmentation of primary care into on-call services, walk-in centres, and the use of pharmacies for first-contact care. Part-time working is partly individual GPs’ responses to workload pressures. Fragmentation of primary care between multiple professionals and organisations is a policy response to workload pressures. Both undermine continuity, contributing to a vicious cycle.

Not all of these changes are inevitable. Throughout the UK, there are general practices, including large general practices, that successfully maintain high continuity of care, despite the same external factors and unfavourable policies.

What are the implications of this for what we should do? Demography and part of the preference for part-time working cannot be changed. But practices might usefully learn from peers that maintain high continuity of care. Can their experience be disseminated and their success emulated? Successful examples of improving continuity include the Health Foundation pilots and, internationally, implementation of a named physician policy in an Israeli health maintenance organisation.^25,26^

The alternative to policies that fragment primary care is to align policies with the aim of improving continuity of care. To this end, the focus should be on delivery of primary care through GPs and general practices. This creates a virtuous cycle of better patient experience, better clinical quality of care, and more sustainable general practices. Policy proposals should be informed by a prior assessment of their likely effects on continuity and, if implemented, should be accompanied by evaluation of their effects on continuity.

## Data

This paper did make use of any data.
